# Spatiotemporal changes in fine particulate matter and ozone in the oasis city of Korla, northeastern Tarim Basin of China

**DOI:** 10.1038/s41598-024-63856-5

**Published:** 2024-06-05

**Authors:** Tayierjiang Aishan, Yaxin Sun, Ümüt Halik, Florian Betz, Asadilla Yusup, Remila Rezhake

**Affiliations:** 1 College of Ecology and Environment, Xinjiang University, Urumqi, 830046 China; 2 Ministry of Education Key Laboratory of Oasis Ecology, Xinjiang University, Urumqi, 830046 China; 3 Faculty of Mathematics and Geography, University of Eichstaett-Ingolstadt, Eichstaett, 85071 Germany; 4 Institute of Ecology, College of Urban and Environmental Sciences, Peking University, Beijing, 100871 China; 5 Affiliated Cancer Hospital of Xinjiang Medical University, Urumqi, 830017 China

**Keywords:** Particulate matter (PM), Ozone, Desert Oasis Cities, Korla, Atmospheric chemistry, Environmental impact

## Abstract

Air pollution is a serious environmental health concern for humans and other living organisms. This study analyzes the spatial and temporal characteristics of air pollutant concentrations, changes in the degree of pollution, and the wavelet coherence of the air quality index (AQI) with pollutants in various monitoring stations. The analysis is based on long-term time series data (January 2016 to December 2023) of air pollutants (PM_2.5_, PM_10,_ and O_3_) from Korla, an oasis city in the northeastern part of the Tarim Basin, China. The concentrations of PM_2.5_, PM_10,_ and O_3_ in Korla showed a cyclical trend from 2016 to 2023; PM_10_ concentrations exhibited all-season exceedance and PM_2.5_ exhibited exceedance only in spring. PM_2.5_ and PM_10_ showed a seasonal distribution of spring > winter > fall > summer; O_3_ concentrations showed a seasonal distribution of summer > spring > fall > winter. Strong positive wavelet coherence between PM and Air Quality Index (AQI) data series suggests that the AQI data series can effectively characterize fluctuating trends in PM concentrations. Moreover, PM_10_ levels IV and VI were maintained at approximately 10%, indicating that sand and dust have a substantial influence on air quality and pose potential threats to the health of urban inhabitants. Based on the results of this study, future efforts must strengthen relative countermeasures for sand prevention and control, select urban greening species with anti-pollution capabilities, rationally expand urban green spaces, and restrict regulations for reducing particulate matter emissions within city areas.

## Introduction

Air pollution is a pressing global environmental concern that has significant impacts on atmospheric visibility, local climate, and the physical and mental health of human beings^1,2^. Air pollution is defined as a condition in which the concentration of specific substances in the air exceeds a certain threshold over a certain period, causing harm to humans or ecosystems^3,4^. Even brief exposure to air pollution raises a noteworthy risk to human health, exacerbating the Global Burden of Disease (GBD) and ultimately contributing to hospitalization and mortality rates^3,5,6^.

Given rapid economic growth, industrialization, and urbanization, the deterioration of urban living environments has become a significant concern. There are up to 3.7 million premature deaths worldwide due to outdoor air pollution^7^. Recent data shows that 7 of the 10 most polluted cities in the world are in China^8^. Air pollution in China exceeds the levels recommended by the WHO^9^ and is the leading cause of death after heart attacks, dietary risks, and smoking^10–12^. Therefore, air quality has garnered significant attention from both the Chinese government and the general public.

Particulate matter (PM) is of great concern due to its small size and large surface area, which makes it easy to adsorb airborne microorganisms, heavy metals, and other toxic substances. PM can penetrate the human body by respiration and settle in the lungs, posing a threat to human health^13^. The 2018 Environmental Performance Index [EPI] shows that the environmental quality of China ranked fourth, including PM_2.5_^14^, China's PM_2.5_ concentrations tend to be well above the National Ambient Air Quality Standards (GB3095-2012 Level 2), which is equivalent to the WHO Interim Target I [IT-1]^4^.

Since China implemented the ‘Ten Measures to Prevent and Control Air Pollution’ in 2013, air quality has improved to some extent; however, the situation remains prominent^15,16^. In recent years, many studies in developed areas of China, such as the Yangtze River Delta, the Pearl River Delta, and the North China Plain, have been conducted on urban air quality based on long-term air pollution data combined with a variety of experimental and modeling methods^17,18^. However, in Xinjiang, a relatively underdeveloped region in northwest China, urban air research has focused on the characteristics, meteorological effects and sources of air pollution in heavily industrialized cities on the northern slopes of the Tianshan Mountains^19^; The impact of the 2015 Taklamakan dust event on the air quality of urban areas in China^20^; Analysis of emission reduction measures and simulation study of PM_2.5_ concentration in Xinjiang's main cotton production area, and study of spatial^21^ and temporal characteristics of air pollution (PM_2.5_, PM_10_, SO_2_, NO_2,_ CO, and O_3_) in surrounding areas caused by a large amount of wind-blown sand from the desert (Taklamakan Desert)^22^.

Korla, an arid zone oasis city, is located adjacent to the Taklamakan Desert and experiences a high number of dust and sandy days throughout the year^23^. As such, it offers the potential to conduct studies related to urban air pollutants and their impact on human health under the influence of sandy and dusty weather. In this study, we utilized long-term daily average data of major PMs—PM_2.5_, PM_10,_ and ozone (O_3_)—in Korla from 2016 to 2023 to quantify the spatiotemporal pattern of each pollutant. Additionally, we investigated the relationship between pollutant concentrations and Air Quality Index (AQI) wavelet coherence (WTC). The results of this study provide valuable scientific references for air quality management in Korla and are of great practical importance for improving air quality conditions, transitioning from natural sand and dust mixed with soot to natural sand and dust, in Korla and other arid oasis urban areas.

## Materials and methods

### Study area

Korla is the capital city of Bayingolin (Bayin'guoleng) Mongolian Autonomous Prefecture (BMAP), China's largest prefecture-level administrative region, is located in Southern Xinjiang. The city sits at the southern foot of the Tianshan Mountains, on the northeastern edge of the Taklamakan Desert, southwest of Lake Bosten. Korla is situated in the transitional zone between the distinct climates of the northern and southern borders; cold and humid air from the north enters the territory of Korla, where it is blocked by the Khora and Kuruk mountains, resulting in a gradual decrease in humidity. It belongs to a warm temperate continental arid climate, characterized by adequate light and heat resources, with an average altitude of 950 m, an average annual rainfall of about 65 mm, and an average annual sunshine of about 2920 hours^23^. The dominant wind direction on the ground throughout the year is northeasterly. The city borders the Taklamakan Desert, experiencing 47.1 days of sand and dust weather per year, leading to severe sand and dust pollution^23,24^. Covering an area of approximately 7,268 km^2^, Korla serves as the gateway to the Middle Road of the ancient Silk Road.

### Methodology

#### Data sources

**Figure 1:**
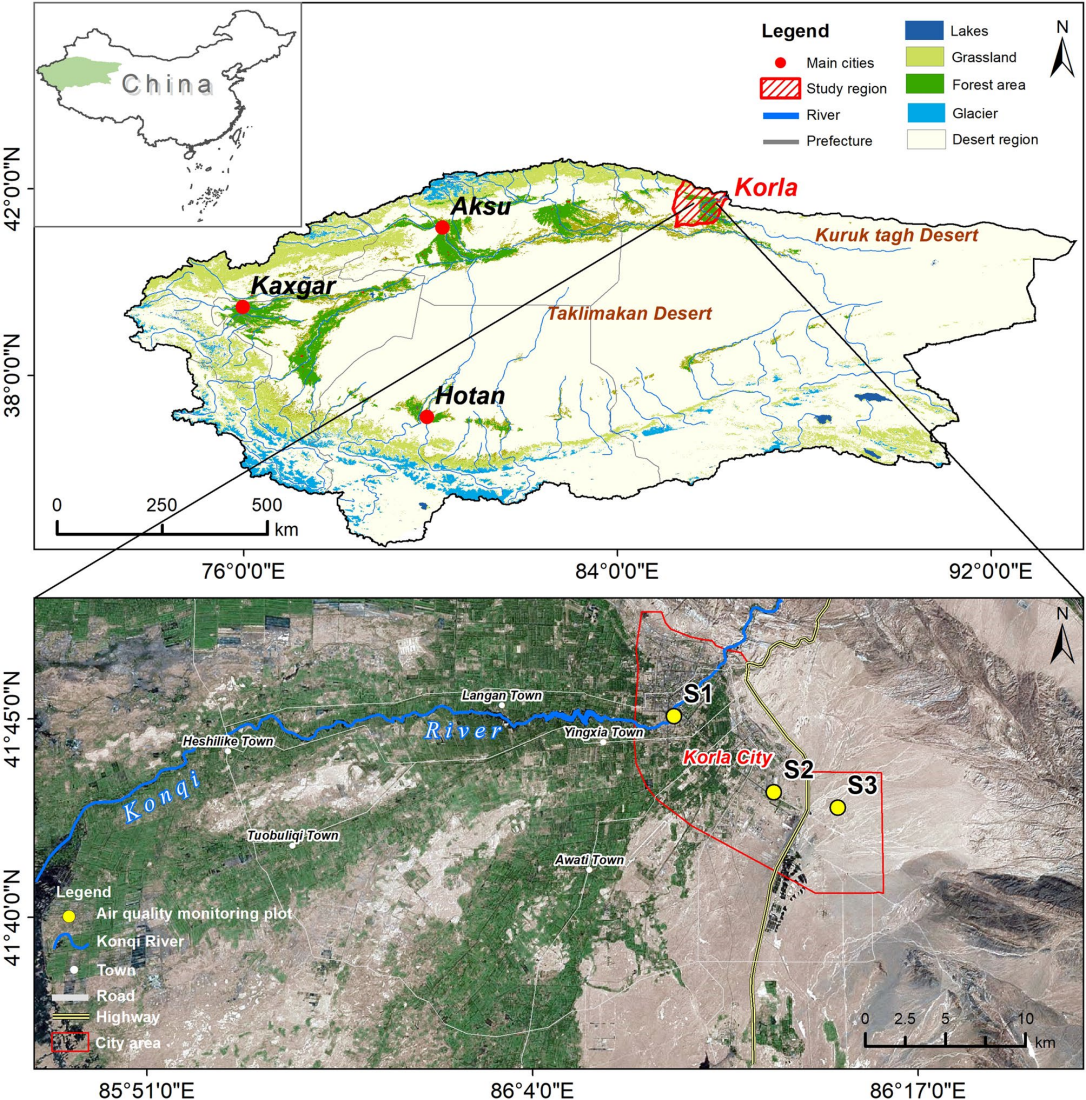
Map of air quality monitoring stations in the study area. The figure is created using ArcMap 10.5 (ESRI Co., Ltd.; www.esri.com).

PM_2.5_, PM_10_, and O_3_ concentration data for Korla between January 1, 2016, and December 31, 2023, were retrieved from the Air Quality Data Network (www.aqistudy.cn/historydata). We selected three state-controlled monitoring stations in Korla that offer uninterrupted data monitoring, namely, Peacock Park (S1), Cotton Spinning Factory (S2), and Economic Development Zone (S3). These stations represent commercial, industrial, and residential areas within the primary urban area of Korla (Fig. 1 and Table 1).

**Table 1:** Overview of air quality monitoring stations in Korla.

Monitoring station	Monitoring station name	Distributed	Geographical location	Area type
S1	Peacock Park	City center	41.75°N、86.15°E	Commercial areas
S2	Cotton Spinning Factory	City center	41.72°N、86.20°E	Industrial areas
S3	Economic Development Zone	City fringe	41.71°N、86.24°E	Residential areas

#### Air quality index

The AQI was calculated according to the Technical Provisions from Ambient Air Quality Index (for Trial Implementation) [HJ 633–2012] and Air Quality Standards" (GB 3095–2012)^25^, as specified below:

1 $${IAQI}_{P}=\frac{{IAQI}_{Hi}-{IAQI}_{L0}}{{BP}_{Hi}-{BP}_{L0}}\left({C}_{P}-{BP}_{L0}\right)+{IAQI}_{L0}$$

where *IAQI*_*P*_ stands for the air quality score of pollutant P, *C*_*P*_ is the concentration of pollutant P, *BP*_*Hi*_ and *BP*_*L0*_ represent the upper and lower limits of the corresponding standard concentration, respectively, *IAQI*_*Hi*_ and *IAQI*_*L0*_ are the air quality sub-indexes corresponding to *BP*_*Hi*_ and *BP*_*L0*_, respectively.

2 $$AQI=\text{max}\left\{{AIQI}_{1},{AIQI}_{2},{AIQI}_{3},\cdots {AIQI}_{n}\right\}$$

where *IAQI* and n are the air quality subindex and pollutant item, respectively. As known from the formula (2), if the air quality sub-index for multi-pollutants exceeds the standard, then *AQI* takes the largest air quality sub-index of pollutants.

All three monitoring sites are categorized as the Class II zone of the ambient air functional area classification (AAFC). Based on the associated requirements, the Class II zone applies to National Secondary Concentration Limits [NSCL]^25^. The 24-h average NSCL limits corresponding to PM_2.5_ and PM_10_ are 75 and 150 µg·m^-3^, respectively; the NSCL for the daily maximum 8-h average concentration of O_3_ is 160 µg·m^-3^.

Based on the AQI levels and corresponding concentration limits of PM_2.5_, PM_10_, and O_3_ in the Technical Provisions on Ambient AQI (for Trial Implementation) [HJ 633–2012], PM_2.5_, PM_10,_ and O_3_ were classified into six levels^26^ (Table 2). The higher the ambient AQI, the more serious the pollution is.

**Table 2:** Ambient air quality level standards and pollutant concentration limits.

Air quality index(AQI)	PM_2.5_ 24-h average/µg m^−3^ concentration limit	PM_10_ 24-h average/µg m^−3^ concentration limit	O_3_ 8-h average/µg m^−3^ concentration limit	Air quality index level	Air quality index category and colors	
0–50	0–35	0–50	0–100	I	Excellent	Green
51–100	36–75	51–150	101–160	II	Good	Yellow
101–150	76–115	151–250	161–215	III	Light pollution	Orange
151–200	116–150	251–350	216–265	IV	Moderately polluted	Red
201–300	151–250	351–420	265–800	V	Heavy pollution	Purple
> 300	> 250	> 420	–	VI	Severe pollution	Maroon

#### Data processing

Origin2021 was used to measure the average concentrations of PM_2.5_, and PM_10_ over 24 h, and O_3_ over 8 h at the three monitoring stations. Additionally, the software was used to visualize the characteristic curves of the spatiotemporal changes of each pollutant, along with the percentage of the pollution level. Additionally, Origin2021 was employed to chart the characteristic curves depicting the spatiotemporal pattern of each pollutant along with the percentage of the pollution level. Differences in PM_2.5_, PM_10,_ and O_3_ data between sites and temporal variations within sites were tested by one-way analysis of variance (ANOVA; significance. level of *P* = 0.05). SPSS 26.0 was used for all statistical analyses.

The Wavelet Coherence function in MATLAB was employed to analyze wavelet coherence between the AQI and PM_2.5_, PM_10_, and O_3_ in Korla from 2016 to 2023. Wavelet analysis is based on Fourier analysis and can reveal the local characteristics of the object under analysis in both the time and frequency domains. Wavelet coherence was applied to examine the correlation between two sequential data points on several timescales. This coherence is interpreted as a correlation coefficient with a value ranging from 0 to 1, where s represents a smoothing parameter. If smoothing is absent, the wavelet coherence is 1. The squared value of the wavelet coherence coefficient ranges from 0 ≤ R^2^ ≤ 1. Values near 0 suggest a poor correlation, whereas values near 1 indicate a robust correlation^27^. Thus, wavelet coherence can be considered a useful approach for assessing the association between specific parameters and time. Using the following equation, the coefficients can be computed from the wavelet energy spectrum^28^:

$${R}_{YZ}^{2}\left(s\right)=\frac{{\left|<{W}^{YZ}>\left(s\right)\right|}^{2}}{{\left|<{W}^{Y}\left(s\right)>\right|}^{2}{\left|<{W}^{Z}\left(s\right)>\right|}^{2}}$$

3 $$\left|{W}_{i}^{YZ}\left(s\right)\right|=\left|{W}_{i}^{Y}\left(s\right)\overline{{W }_{i}^{Z}\left(s\right)}\right|$$

where *Y* and *Z* are the data sequences; *R*^*2*^_*YZ*_(s) represents the wavelet coherence coefficient; *W*_*i*_^*YZ*^(s) stands for the wavelet cross-spectrum of the data sequence *YZ*; *W*_*i*_^*Y*^ and *W*_*i*_^*Z*^ are the wavelet coefficients for the data sequences *Y* and *Z*, respectively; and " <  > " indicates the smooth function of the wavelet energy spectrum. Detailed computations were conducted using the wavelet toolbox in MATLAB.

## Results and discussion

### Characteristics of spatial and temporal variations in air pollutant concentrations

**Figure 2:**
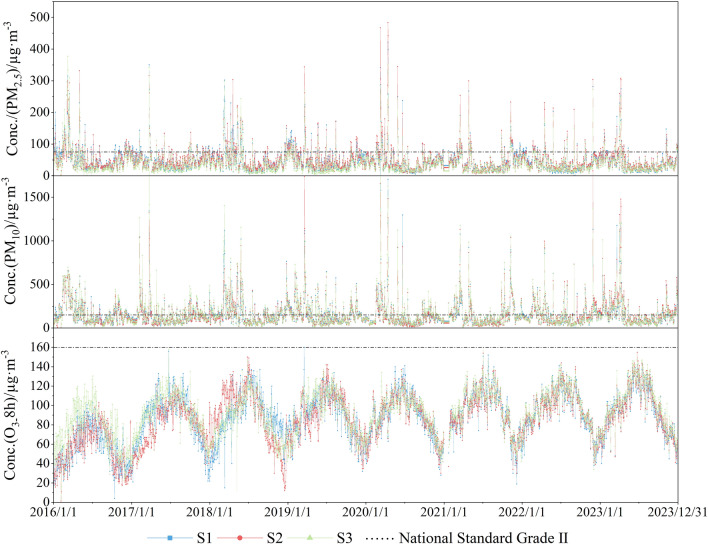
Variation trends in air pollutant concentrations in Korla from 2016 to 2021.

Figure 2 illustrates a nearly identical concentration trend between the air pollutants PM_2.5_ and PM_10_ in Korla from 2016 to 2023, showing an "L" cycle with an annual peak occurring from January to May and a sustained peak in PM_2.5_ concentration changes during this period (winter and spring), particularly evident in 2018. The concentration levels consistently peak, with maximum concentration values reaching up to 500 µg·m^-3^; PM_10_ also exhibits persistent peaks during the same period, with longer peak hours compared to previous years. In contrast, variations in PM_2.5_ and PM_10_ concentrations from June to December (summer and fall) show no significant fluctuations, and the pattern of change remains relatively steady.

The eastern section of Korla is primarily composed of the Taklamakan Desert. During spring and summer months, gusty winds prevail, facilitating the transportation of sand and dust particles into the air, resulting in elevated levels of atmospheric PM^29,30^. Higher atmospheric PM levels during winter months have two main contributing factors. First, the prolonged period of winter heating in the city leads to the accumulation of PM from coal combustion, thereby increasing the concentration^30,31,32^. Second, the winter climate in oasis cities located within arid regions is cold and dry, characterized by stable atmospheric structures that promote inversions. These inversions hinder the dispersion and dilution of atmospheric pollutants^33^.

Additionally, the high humidity and precipitation in summer promote the wet deposition of PM, resulting in low PM_2.5_ and PM_10_ concentrations during this season. Furthermore, the stronger solar radiation in summer enhances atmospheric turbulence movement, contributing to the diffusion and dilution of atmospheric PM^34^. The concentration of O_3_ exhibits an inverted "V" pattern, with apex concentration occurring between April and September (C_max_ ≈ 150 µg m^−3^), and the lowest concentration levels observed in January (C_min_ ≈ 20 µg m^−3^). Solar radiation intensity significantly influences photochemical reactions, with variations in air temperature can affecting radiation intensity. Hence, both high temperatures and radiation promote O_3_ production^34,35^.

China's air quality standards are based on the WHO air quality guidelines for PM and transitional objectives that measure changes in 24-h concentrations^25^. These standards require Class II areas of the AAFC to adhere to Class II standards. The particulate pollutant PM_10_ exceeded the standard in Korla in almost all seasons, and PM_2.5_ exceeded the standard in spring. The frequent sand and dust weather during spring suggests that the impact of sand and dust on PM concentrations is significant, highlighting the concern about atmospheric particulate pollution in Korla. Additionally, the air pollutant O_3_ exceeded the national secondary standard only on individual days during 2016–2023, indicating that Korla has taken actions to control total pollutant emissions, strengthen pollution management, control emissions from motorized vehicles, adjust the energy use structures, and achieve effective results. Consequently, air pollution in Korla has gradually shifted from mixed sources (natural sand, dust, and soot) to natural sand and dust^1^.

### Seasonal characteristics of PM_2.5_, PM_10_, and O_3_

The main traffic corridors of North and South Xinjiang, National Highway 218 and National Highway 314, pass around Korla and provide complex sources of PM. PM_2.5_, and PM_10_ at different monitoring stations in Korla from 2016 to 2023 were the highest in spring, lowest in summer, and intermediate and comparable in fall and winter, with mean concentration values changing from spring > winter > fall > summer (Table 3). Based on the mean PM_2.5_ concentration at the three monitoring stations, it is evident that S2 had the highest concentration, followed by S1 and S3. The highest concentration of 63.8 ± 2.2µg m^−3^ was observed in spring at station S2, while the lowest value of 23.4 ± 0.6 µg m^−3^ occurred in summer at station S3. However, pollutant emissions resulting from energy consumption during industrial production in industrial areas contribute more to PM_2.5_ than residential and motorized trips^4^.

**Table 3:** Seasonal changes in PM_2.5_, PM_10,_ and O_3_ concentrations at different monitoring sites in Korla city during 2016–2021.

		2016	2017	2018	2019	2020	2021	2022	2023	Annual mean
Conc.(PM_2.5_) /µg m^−3^										
S1	Spring	78.2 ± 6.4Ab	49.5 ± 4.9Bb	82.5 ± 6.1Aa	50.4 ± 4.6Cb	68.9 ± 8.3ABa	51.2 ± 4.2Ca	43.2 ± 3.3Cb	53.7 ± 5.3BCa	59.7 ± 2.0
	Summer	33.5 ± 1.8ABd	29.8 ± 2.0Bc	28.1 ± 2.7BCc	37.8 ± 2.7Ac	23.6 ± 2.7CDd	18.8 ± 1.3Db	23.1 ± 1.8CDd	21.5 ± 0.9Dc	27.0 ± 0.8
	Autumn	44.0 ± 2.4ABc	48.7 ± 2.2Ab	35.8 ± 1.7Cc	39.8 ± 2.2BCc	38.8 ± 1.7BCc	46.2 ± 3.4ABa	33.3 ± 3.5Cc	35.6 ± 2.4Cb	40.3 ± 0.9
	Winter	83.4 ± 3.0Aa	60.6 ± 2.2Ca	56.5 ± 2.5CDb	71.7 ± 2.9Ba	54.5 ± 1.8CDb	50.0 ± 1.9Da	58.2 ± 1.9Cb	56.2 ± 2.4CDa	61.4 ± 0.9
S2	Spring	78.6 ± 7.4ABa	49.7 ± 4.5Ca	77.5 ± 5.9ABa	58.4 ± 5.5Ca	81.8 ± 9.7Aa	55.4 ± 5.0Ca	47.3 ± 4.0Cab	61.6 ± 5.9BCa	63.8 ± 2.2
	Summer	35.5 ± 1.9ABb	38.8 ± 2.0Ab	34.7 ± 2.6ABc	39.2 ± 2.5Ab	25.2 ± 2.5CDc	21.6 ± 1.2Dc	29.5 ± 2.1BCc	27.4 ± 1.0CDc	31.5 ± 0.8
	Autumn	42.7 ± 1.8BCb	53.4 ± 2.2Aa	40.1 ± 1.8BCc	48.1 ± 2.7ABb	41.7 ± 2.0BCb	48.1 ± 4.2ABab	40.4 ± 4.0BCb	38.4 ± 2.7Cb	44.1 ± 1.0
	Winter	69.0 ± 3.0Ba	54.2 ± 1.9CDEa	53.1 ± 2.4BCDb	63.4 ± 2.7Ba	54.9 ± 2.4Eb	43.1 ± 1.7DEb	53.8 ± 2.1BCa	56.0 ± 2.5Aa	55.9 ± 0.9
S3	Spring	78.5 ± 8.1Aa	42.0 ± 4.9BCa	75.5 ± 6.1Aa	39.7 ± 4.4Cb	57.5 ± 7.0Ba	45.4 ± 4.0BCa	41.5 ± 3.2BCa	50.0 ± 4.9BCa	53.8 ± 2.0
	Summer	18.3 ± 1.4Cc	26.8 ± 1.8Ab	24.1 ± 2.7ABc	25.1 ± 2.1ABc	23.9 ± 1.8ABc	20.7 ± 1.1BCc	25.4 ± 1.9ABb	23.0 ± 0.6ABCc	23.4 ± 0.6
	Autumn	33.4 ± 1.7BCDb	41.4 ± 2.2Aa	26.9 ± 1.5Dc	32.1 ± 1.9CDc	36.0 ± 1.7ABCb	39.7 ± 3.5ABab	32.2 ± 1.8CDb	32.6 ± 1.8CDb	34.3 ± 0.8
	Winter	67.2 ± 3.9Aa	43.8 ± 1.8Ca	41.4 ± 2.2CDb	54.1 ± 2.5Ba	45.4 ± 1.7Cb	35.3 ± 1.8Db	44.8 ± 1.8Ca	46.3 ± 2.2Ca	47.3 ± 0.9
Conc.(PM_10_) /µg m^−3^										
S1	Spring	267.3 ± 17.1ABa	133.6 ± 18.5Da	296.5 ± 23.6Aa	201.1 ± 21.1BCDa	267.0 ± 37.6ABa	184.9 ± 18.7CDa	181.4 ± 15.6CDa	241 ± 26.2ABCa	221.6 ± 8.4
	Summer	90.9 ± 5.9Bc	91.3 ± 5.6Bb	95.2 ± 9.2Bc	121.7 ± 10.0Ab	82.9 ± 14.1Bb	71.8 ± 3.7Bc	86.4 ± 7.7Bc	75.0 ± 3.0Bc	89.7 ± 3.0
	Autumn	135.3 ± 7.9BCb	155.0 ± 7.3Ba	120.1 ± 6.0Cc	137.7 ± 8.7BCb	126.9 ± 5.8BCb	197.3 ± 18.7Aa	122.1 ± 14.2BCb	123.8 ± 8.4BCb	139.8 ± 3.8
	Winter	206.2 ± 16.0Aa	156.8 ± 14.0BCa	176.5 ± 11.1ABb	198.6 ± 12.0Aa	121.3 ± 10.8Db	134.4 ± 6.5CDb	174.2 ± 8.4ABa	208.3 ± 10.8Aa	172.0 ± 4.2
S2	Spring	261.8 ± 17.9ABa	132.8 ± 18.9Da	264.6 ± 20.0ABa	210.2 ± 25.2ABCb	244.1 ± 32.9ABa	197.4 ± 19.7BCDa	168.3 ± 16.6CDa	277.2 ± 29.0Aa	219.5 ± 8.4
	Summer	80.2 ± 4.7BCDd	92.0 ± 5.7ABb	94.8 ± 8.9ABc	108.4 ± 9.4Ab	69.0 ± 11.0CDc	58.8 ± 3.3Dc	98.4 ± 9.6Ab	83.3 ± 3.8BCc	85.6 ± 2.7
	Autumn	130.7 ± 8.1BCc	150.2 ± 6.8BCa	114.9 ± 6.0Cc	138.3 ± 9.3BCa	127.5 ± 6.4BCb	196.4 ± 19.4Aa	156.6 ± 21.6Ba	146.9 ± 10.0BCb	145.2 ± 4.4
	Winter	185.7 ± 16.9Bb	145.0 ± 13.9Aa	159.9 ± 10.8Bb	192.5 ± 12.3Aa	117.0 ± 10.5BCbc	134.5 ± 8.0Bb	180.7 ± 9.8BCa	242.2 ± 13.6Aa	169.7 ± 4.5
S3	Spring	273.3 ± 17.9ABCa	201.8 ± 28.0CDa	337.2 ± 28.2Aa	205.1 ± 20.2BCDa	282.8 ± 37.2ABa	212.3 ± 21.5BCDa	183.4 ± 16.4Da	248.3 ± 26.2BCDa	243.0 ± 9.0
	Summer	63.7 ± 5.6Cc	97.7 ± 6.7ABb	90.3 ± 9.7ABc	109.4 ± 10.6Ab	89.8 ± 9.4ABc	74.6 ± 4.2BCc	90.4 ± 8.8ABb	75.1 ± 3.5BCd	86.4 ± 2.8
	Autumn	153.0 ± 10.2BCb	184.7 ± 9.6Ba	117.6 ± 6.0Cc	144.8 ± 10.1Cb	154.6 ± 8.3BCb	221.5 ± 20.0Aa	120.4 ± 17.9Cb	131.7 ± 8.7Cc	153.5 ± 4.5
	Winter	189.4 ± 17.5Ab	167.4 ± 18.3ABa	181.0 ± 10.2Ab	195.6 ± 13.8Aa	127.9 ± 12.3Bbc	137.1 ± 8.5Bb	165.4 ± 9.7ABa	204.0 ± 13.9Ab	170.5 ± 4.8
Conc.(O_3_) /µg m^−3^										
S1	Spring	61.6 ± 1.7Da	97.8 ± 1.3Bb	91.9 ± 1.8Cb	99.0 ± 1.4Bb	99.0 ± 1.6Bb	98.9 ± 1.4Ba	103.6 ± 1.2Bb	103.8 ± 1.3Ab	94.4 ± 0.7
	Summer	75.7 ± 1.3Ea	105.2 ± 1.4Ca	113.1 ± 1.5Ba	109.4 ± 1.2Ba	110.4 ± 1.4Ba	93.5 ± 4.9Da	113.5 ± 1.7Ba	121.8 ± 1.2Aa	105.4 ± 0.9
	Autumn	45.6 ± 1.8Cb	83.6 ± 1.8ABc	85.3 ± 1.2Ac	80.8 ± 1.9ABc	78.9 ± 1.3Bc	83.4 ± 2.6ABb	85.3 ± 2.7Ac	82.5 ± 1.5ABc	78.2 ± 0.8
	Winter	36.9 ± 1.0Db	55.2 ± 1.7Cd	58.3 ± 1.8Cd	68.5 ± 1.5ABd	66.6 ± 1.9Bd	69.7 ± 2.0ABc	72.4 ± 1.5Ad	72.5 ± 1.7Ad	62.3 ± 0.7
S2	Spring	57.3 ± 1.9Eb	77.8 ± 1.6Dc	109.3 ± 1.6Aa	90.5 ± 1.5Cb	99.8 ± 1.4Ba	97.1 ± 1.4Bb	99.7 ± 1.1Ba	101.4 ± 1.5Bb	91.6 ± 0.8
	Summer	77.6 ± 1.1Da	99.9 ± 1.1Ca	111.7 ± 1.7Ba	112.6 ± 1.3Ba	99.8 ± 2.8Ca	113.8 ± 1.5Ba	114.7 ± 1.3Bb	124.7 ± 1.3Aa	106.8 ± 0.7
	Autumn	47.5 ± 1.8Dc	86.0 ± 1.0Bb	65.2 ± 1.5Cb	86.5 ± 1.9Bb	81.8 ± 1.3Bb	85.1 ± 2.1Bc	93.6 ± 1.8Ab	84.0 ± 1.5Bc	78.7 ± 0.8
	Winter	33.2 ± 1.4Dd	53.3 ± 1.3Cd	65.5 ± 3.1Bb	69.5 ± 1.4ABc	65.4 ± 1.9Cc	53.4 ± 3.6Cd	73.6 ± 1.5Aa	72.6 ± 1.5Ad	60.8 ± 0.9
S3	Spring	86.9 ± 1.8Da	100.4 ± 1.3Bb	91.3 ± 1.5Cb	107.2 ± 1.1Ac	100.8 ± 1.3Bb	104.9 ± 1.5Ab	105.2 ± 1.1Ab	107.1 ± 1.4Ab	100.5 ± 0.6
	Summer	75.7 ± 4.2Eb	110.0 ± 1.2Da	110.6 ± 1.4Da	111.4 ± 1.2CDa	105.9 ± 1.1Da	116.3 ± 1.3BCa	117.6 ± 1.1Ba	127.3 ± 1.2Aa	109.4 ± 0.8
	Autumn	61.4 ± 1.8Ec	88.6 ± 1.3BCc	73.4 ± 1.6Dc	83.7 ± 1.7Cc	84.7 ± 1.4BCc	87.0 ± 2.1BCc	94.9 ± 1.8Ac	89.1 ± 1.6Bc	82.9 ± 1.6
	Winter	53.4 ± 1.6Dd	68.6 ± 2.2BCd	63.4 ± 1.4Cd	67.4 ± 1.1BCd	69.9 ± 2.0Bd	55.4 ± 3.6Dd	76.6 ± 1.6Ad	76.8 ± 1.4Ad	66.3 ± 0.8

Mean ± standard error, different capital letters indicate statistically significant differences between seasons, and lowercase letters indicate statistically significant differences between study years (*P* < 0.05).

The pollutant PM_2.5_ concentrations at each site indicate an overall decrease from 2016 to 2023. This trend was particularly distinguishable in winter and spring. As shown in Table 3, the analysis of seasonal differences (uppercase letters) and interannual differences (lowercase letters) in PM_2.5_ at the three monitoring stations revealed that no significant differences were found between spring and winter within the stations and that the differences between summer and fall were not significant; however, significant differences were found between spring and winter and between summer and fall (*P* < 0.05). There was no significant difference statistically (*P* > 0.05) in the annual mean PM_2.5_ among the study years at each site. The changes in the mean PM_10_ concentrations at the three monitoring sites were opposite to those of PM_2.5_, showing S3 > S1 > S2, but all their peaks occurred in the spring, at 243.0 ± 9.0, 221.6 ± 8.4, and 219.5 ± 8.4 µg m^−3^, respectively; the minimum value was 85.6 ± 2.7 µg m^−3^ (in the summer at site S2). However, PM_10_, a type of particulate pollutant, displayed comparable changes to PM_2.5_, also showing significant variance among seasons (i.e., winter, spring, summer, and fall). However, this variation was less noticeable between the years (*P* > 0.05). As previously mentioned, the study area demonstrates significant seasonal fluctuations in atmospheric particulate pollutants, specifically PM_2.5_ and PM_10_. Potential factors contributing to elevated levels during the winter months include the use of coal-fired heating for residential purposes, while the presence of dusty weather in spring may also play a role. The gradual decrease in PM_2.5_ and PM_10_ concentrations from 2016 to 2023 indicates the significance of the control measures implemented for air pollutants in Korla.

Changes in O_3_ pollutant concentration at all sites from 2016 to 2023 indicate a gradual overall increase with a sharper incline in the spring and summer seasons (approximately a 30 µg m^−3^ increase), whereas the increase during fall and winter is slightly lower than that during the former seasons. Additionally, the monitoring sites exhibited a seasonal distribution of O_3_ concentrations, with the highest concentration documented in the summer at site S3 in 2023 (C_max_ ≈ 127.3 µg m^−3^), and the lowest concentration of 33.2 µg m^−3^ recorded at site S2 in 2016 during the fall and winter seasons. The O_3_ concentrations at each site showed a significant difference between seasons (*P* < 0.05). Interannual differences in O_3_ concentrations were observed only between individual years (e.g., significant differences in concentrations between 2016 and other study years at each station; however, there was no significant variability in O_3_ concentrations between each year from 2017 to 2023). A study characterizing the seasonal distribution of O_3_ concentrations revealed that high temperatures and intense high-temperature radiation during summer, particularly in Korla, which is close to the desert, significantly affect the variation in O_3_ concentrations^36^. Conversely, cooler weather and lower radiation levels during winter did not promote O_3_ formation.

### Wavelet-based analysis of coherence between AQI with PM_2.5_, PM_10_, and O_3_

The relationship between AQI and PM_2.5_, PM_10_, and O_3_ was examined using WTC. WTC images display time on the horizontal axis and frequency on the vertical axis^27^. The frequency range covered high frequencies (0–16), intermediate frequencies (16–128), and low frequencies (128–512). The WTC spectral intensity was defined using a color-coded scheme (blue and red indicate low and high intensity, respectively). Our analysis focused on the cone of influence (COI), which represents the colored region where K is not affected by edge effects in the wavelet spectrum; in contrast, the region included in the thick line represents a 95% confidence interval relative to the red noise [i.e., significance based on Monte Carlo simulations]^37–40^.

The arrows in the wavelet coherence plot demonstrate the relationships and causal processes between AQI and PM_2.5_, PM_10_, and O_3_. The left ( ←) and the right ( →) arrows denote positive and negative correlations, respectively^40^. In addition, the rightward and downward (or leftward and upward) directions depict a cause-effect relationship between the first and second parameters, while the rightward and upward (or leftward and downward) directions indicate a cause-and-effect relationship between the second and first parameters^27^.

**Figure 3:**
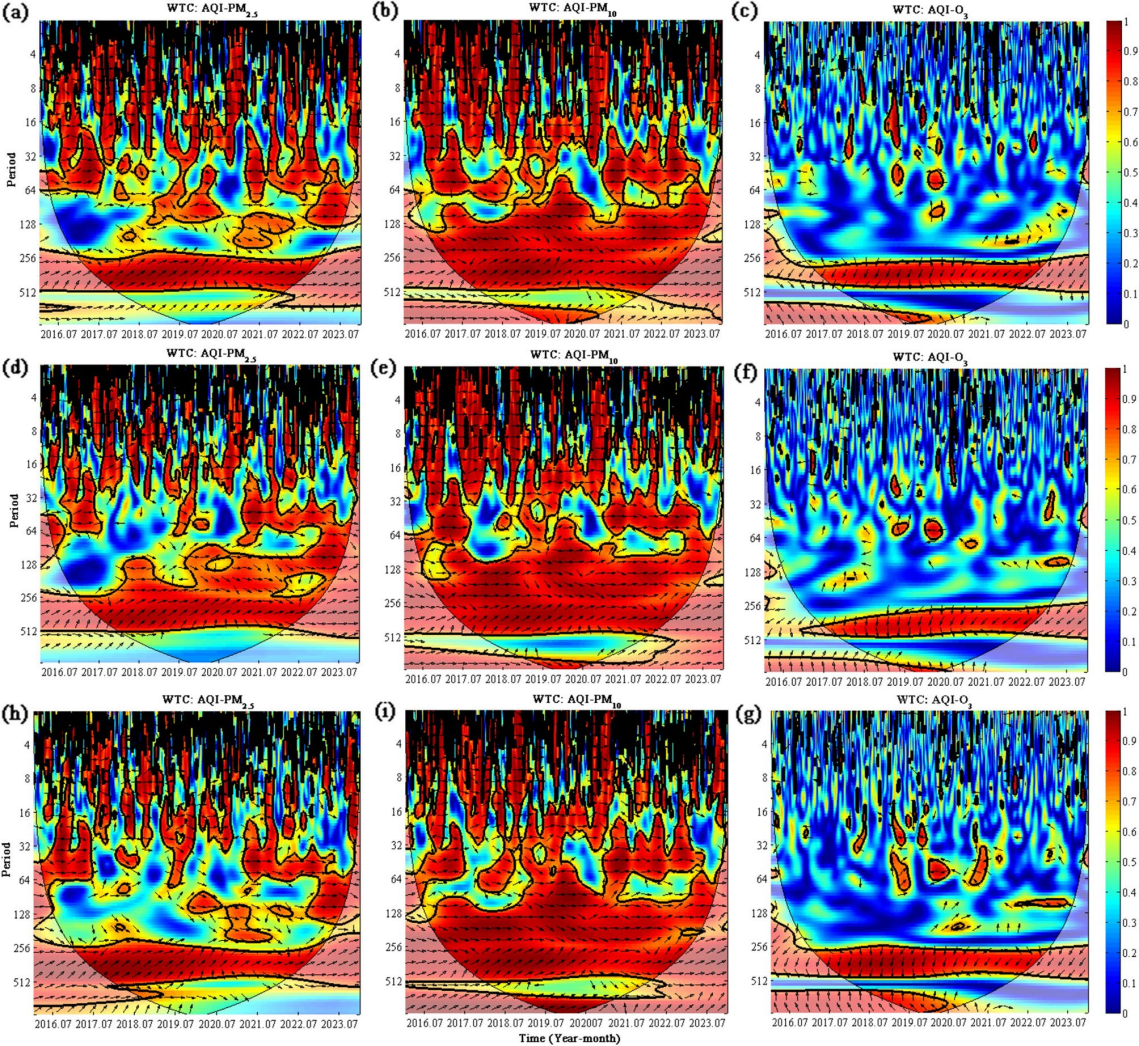
Wavelet coherence of AQI with S1 site: PM_2.5_ (**a**), PM_10_ (**b**) and O_3_ (**c**), S2 site: PM_2.5_ (**d**), PM_10_ (**e**) and O_3_ (**f**), and S3 site: PM_2.5_ (**h**), PM_10_ (**i**) and O_3_ (**g**).

As shown in Fig. 3a, from 2016 to 2023, AQI and PM_2.5_ pollutant levels at site S1 show a positive correlation at various frequencies, including the medium–high (0–64) and low (256–512) frequency ranges. Most arrows depict a rightward and upward trend, indicating that the PM_2.5_ pollutant sequence affects the AQI sequence. There is a short-term positive correlation between AQI and PM_2.5_ in the 64–128 frequency range from 2018 to 2022. As shown in Fig. 3b, there is a long-term positive correlation between AQI and PM_10_ over the range 0–512 (high–low) from 2016–to 2023, with a weaker correlation observed at intermediate-frequencies (16–64) compared with the high and low-frequency ranges. Figure 3c illustrates uninterrupted wavelet coherence between AQI and O_3_ at site S1 at low frequencies (256–512). However, a more pronounced hysteresis effect was observed. Furthermore, the wavelet coherence distributions at intermediate- and high-frequency periods (0–128) are sporadic and lack informative value.

Figure 3d displays the WTC results for PM_2.5_ and AQI at site S2. A positive correlation is observed, as evidenced by the majority of arrows pointing to the right. Specifically, the high-frequency (0–16) and low-frequency (256–512) ranges show significant coherence. In addition, in the intermediate-frequency (16–64) range, a positive correlation between AQI and pollutant PM_2.5_ exists only for short periods, and the correlation is weak (e.g., 2016–2017 and 2020–2021). Over the 2016–2023 period, a positive correlation between AQI and PM_10_ in the 0–512 range was observed with a high-to-low trend. This correlation was short-term, isotropic, and limited to the intermediate-frequency range (16–64; Fig. 3e). Furthermore, the robust correlation between AQI and PM_10_ during these periods demonstrated that the AQI data accurately portrayed the varied trends of PM_10_ pollutants. A significant downward leftward arrow indicates a continuous wavelet negative correlation between AQI and O_3_ at S2, specifically in the low-frequency range (256–512; Fig. 3f).

From 2016 through 2023, the majority of arrows in the medium–high (0–64) and low (256–512) frequency ranges indicate a positive correlation between AQI and PM_2.5_ at the S3 site and an in-phase relationship between the two variables (Fig. 3h). However, the correlation between AQI and PM_2.5_ is less significant in the low-intermediate-frequency range (64–128). Figure 3i shows the cross-correlations between AQI values of S3 and PM_10_. Over the long term (2016–2023), a positive correlation between AQI and PM_10_ was shown at various frequencies and scales. This link is notable for frequencies ranging from 0 to 16 (high frequencies) and 128 to 512 (low frequencies). Furthermore, there are indications that AQI and PM_10_ (upper and lower right arrows) exhibit mutual causality during these periods. The arrows on both sides are within the range of 16–64 (intermediate-frequency; Fig. 3g), indicating irregular fluctuations in the wavelet coherence between the two. In addition, there is continuous wavelet coherence between AQI and O_3_ within the low-frequency (256–512) range; however, there is a notable hysteresis effect between the two (downward arrows on the left).

In summary, wavelet coherence analysis showed that AQI at every monitoring station shared continuous wavelet coherence with both PM_2.5_ and PM_10_ during various periods and displayed the same phase, indicating a substantial correlation between AQI and the pollutants. However, long-term wavelet coherence between AQI and O_3_ is fragmented, and a hysteresis effect is apparent. Our results show that utilizing AQI values to examine ozone fluctuation traits is not suitable. Instead, AQI data efficiently characterize PM_10_ fluctuations and underpin reliable data for air pollution management and health risk assessment in the region.

### Air pollution levels of PM_2.5_, PM_10_, and O_3_

**Figure 4:**
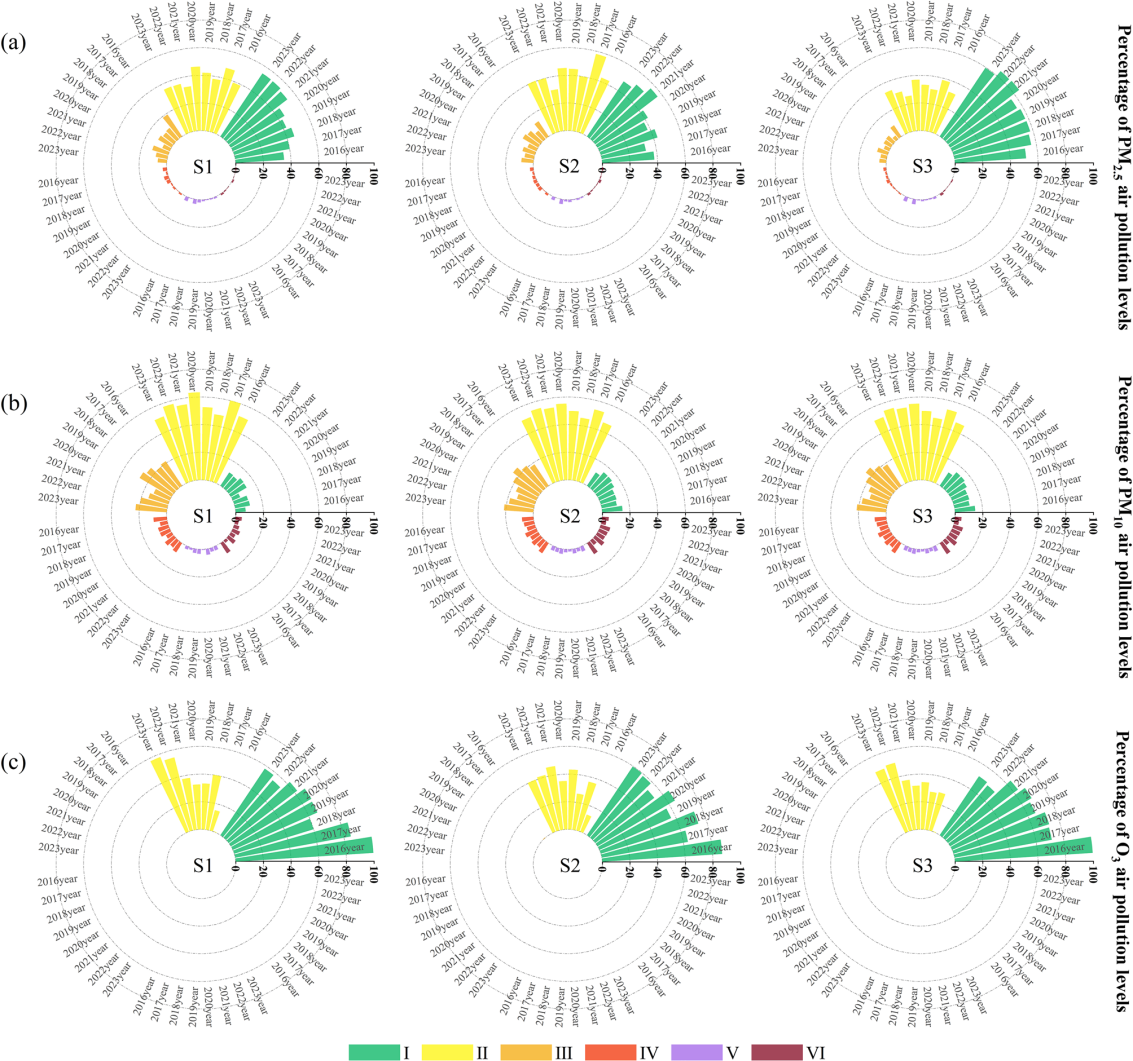
Percentages of days for each PM_2.5_ (**a**), PM_10_ (**b**), and O_3_ (**c**) pollution level at three monitoring sites in Korla.

Table 2 and Fig. 4a indicate that between 2016 and 2023, the percentage of days when the air quality of PM_2.5_ at the three air monitoring stations in Korla was class I (excellent), accounted for one-third of the annual number of days, suggesting an improvement in regional air quality at these stations, with this trend increasing annually. However, the percentage of days with Class II (good) and Class III (slightly polluted) air quality at the three monitoring stations exhibited an undulating trend. The number of days with regional air quality classes I to III at the three monitoring stations reached over 90% of the total number of days in the year, indicating lower pollution levels by PM_2.5_ for residents of Korla. However, the percentage of IV (moderately polluted) and V (heavily polluted) days at site S2 was slightly higher than that at sites S1 and S3, possibly due to its location in an industrial area.

Analysis of the air quality class share of PM_10_ from 2016 to 2023 showed that class II days accounted for 50%–60% of the annual number of days at all three monitoring stations, with the share of air quality class II at site S1 exceeding 60% in 2020 (Fig. 4b). During the period 2016–2023, the proportion of Class III air quality days at the three monitoring stations displayed a trend of increasing, then decreasing, and then increasing; however, the proportion of Class I air quality changed in the opposite direction, indicating an improving air quality trend in the three regions of Korla. In addition, the percentage of days with air quality levels IV to VI at the three monitoring stations in Korla ranged from 0 to 15% of the number of days in the year. The percentage of days with moderate and heavy pollution showed a trend of decreasing, then increasing, and then decreasing, while the percentage of days with severe pollution showed a slight decrease from year to year. Moreover, the trend of air quality class percentage of particulate pollutant PM_10_ at the three stations is almost the same, indicating stable change in PM_10_ in Korla during 2016–2023. Natural sand and dust contribute to an increase in PM_10_, reflecting that natural sand and dust are the main factors causing particulate pollution in the city.

Figure 4c indicates that compared with PM_2.5_ and PM_10_, the air quality class of O_3_ in Korla is good, with air quality maintained at excellent (I) and good (II) levels throughout the year. The maximum percentages of air quality class I for O_3_ at the three monitoring stations all appeared in 2016 and were in the order of S1 > S2 ≈ S3. This may be because the S1 site is located in the center of Korla, near the Peacock River, where several O_3_ precursors (e.g., some oxygenated organic compounds) can be purified by moisture, indirectly reducing the potential for O_3_ production^41^. Although the overall O_3_ level in Korla is excellent, the proportion of O_3_ class I showed a decreasing trend every year from 2016 to 2023, which may be due to an increase in atmospheric O_3_ caused by the volatile organic compounds (VOCs) emitted from industrial activities and motorized vehicles accompanying the rapid development of the city^42^.

When air pollutant levels of PM_2.5_ and PM_10_ reach levels IV–V, they potentially affect the heart and respiratory system of healthy individuals, significantly exacerbating symptoms in cardiorespiratory patients, reducing exercise tolerance, and inducing symptoms in the general population. At severe pollution levels (class VI), exercise tolerance in healthy individuals is markedly reduced, with noticeable and severe symptoms, leading to the early onset of certain diseases^43,44^. Prolonged exposure to high O_3_ concentrations damages the human respiratory system and contributes to the development of eye diseases and other related illnesses^45^. Therefore, relevant city departments should actively implement measures for sand prevention and control, such as adopting the straw checkboard engineering model, implementing policies to convert farmland to forests and grasslands, promoting tree planting initiatives, and establishing a monitoring and early-warning system for sand and dust intensity using modern technologies such as satellite remote sensing, radar, and airborne sounding, etc^46,47^. Simultaneously, these measures should aim to reduce sources of particulate pollutant emissions, decrease PM concentrations, and strengthen management and control measures for O_3_ to safeguard residents' health, preserve ecological safety, and optimize the environmental ecosystem.

## Conclusion

From 2016 to 2023, the concentrations of PM_2.5_ and PM_10_ in Korla City exhibit a seasonal distribution of spring > winter > autumn > summer, characterized by low concentrations in summer and autumn and high concentrations in winter and spring. In contrast, O_3_ demonstrates a seasonal distribution of summer > spring > autumn > winter, displaying an opposite trend to that of particulate matter concentration. Korla City's proximity to the Taklamakan Desert results in dusty weather during spring, contributing to high atmospheric concentrations of particulate matter. The AQI, PM_2.5_, and PM_10_ at each monitoring site in Korla City exhibit continuous wavelet coherence at different periods, indicating significant connections between AQI and pollutants. However, the long-term wavelet coherence between AQI and O_3_ is fragmented, with a visible lag effect, making it inappropriate to utilize AQI values to examine the properties of ozone oscillations in Korla. Conversely, AQI effectively defines PM_2.5_ and PM_10_ fluctuations, providing credible data support for regional air pollution control and health risk assessments. Furthermore, the air quality of O_3_ in Korla has consistently been outstanding, with air pollution level ratings of PM_2.5_ and PM_10_ in the range of I-III accounting for more than 90% of the total number of days in the year, indicating overall satisfactory air quality. However, from 2016 to 2023, the number of days with air pollution level ratings of PM_10_, a particulate matter, at the three monitoring stations reaching Class VI (severe pollution) accounted for 5–10% of the total number of days in the year, highlighting the prominent impact of sand and dust and its potential threat to residents' health. Consequently, relevant city authorities should prioritize strengthening sand prevention and control policies and minimizing particulate emissions.

This study enriches research on urban air pollution in arid land oasis cities in Northwest China. Recent prevention and control efforts of urban pollutants have led to significant improvements in air quality in Korla. Urban planners, ecologists, meteorologists, decision-makers, and other stakeholders in the region must collaborate, and continue to implement proactive measures to manage sand and dust effectively, strengthen sand prevention and control policies, select greenery species with strong anti-pollution capabilities, increase urban green coverage, and reduce PM emissions and concentrations. By doing so, they can effectively reduce and mitigate urban air pollution, thereby enhancing air quality for city residents.

## Data Availability

The datasets used and/or analyzed during the current study are available from the corresponding author on reasonable request.
